# The Universal Non-Neuronal Nature of Parkinson’s Disease: A Theory

**DOI:** 10.5195/cajgh.2016.231

**Published:** 2016-06-01

**Authors:** André X. C. N. Valente, Altynai Adilbayeva, Tursonjan Tokay, Albert A. Rizvanov

**Affiliations:** 1 Center for Neuroscience and Cell Biology, University of Coimbra, Cantanhede, Portugal;; 2 Biocant - Biotechnology Innovation Center, Cantanhede, Portugal;; 3 Institute of Fundamental Medicine and Biology, Kazan Federal University, Kazan, Russia;; 4 National Laboratory Astana, Nazarbayev University, Astana, Kazakhstan

**Keywords:** Parkinson’s disease

## Abstract

Parkinson’s disease (PD) is one of the most common neurodegenerative disorders, yet the etiology of the majority of its cases remains unknown. In this manuscript, relevant published evidence is interpreted and integrated into a comprehensive hypothesis on the nature, origin, and inter-cellular mode of propagation of sporadic PD. We propose to characterize sporadic PD as a pathological deviation in the global gene expression program of a cell: the PD expression-state, or PD-state for short. A universal cell-generic state, the PD-state deviation would be particularly damaging in a neuronal context, ultimately leading to neuron death and the ensuing observed clinical signs. We review why ageing associated accumulated damage caused by oxidative stress in mitochondria could be the trigger for a primordial cell to shift to the PD-state. We propose that hematopoietic cells could be the first to acquire the PD-state, at hematopoiesis, from the disruption in reactive oxygen species homeostasis that arises with age in the hematopoietic stem-cell niche. We argue that cellular ageing is nevertheless unlikely to explain the shift to the PD-state of all the subsequently affected cells in a patient, thus indicating the existence of a distinct mechanism of cellular propagation of the PD-state. We highlight recently published findings on the inter-cellular exchange of mitochondrial DNA and the ability of mitochondrial DNA to modulate the cellular global gene expression state and propose this could form the basis for the inter-cellular transmission of the PD-state.

Parkinson’s disease (PD) is a common neurodegenerative disorder associated with old age. The number of worldwide affected individuals is estimated at 7 to 10 million.^1^ With population ageing, particularly in developing countries^2^, this number is expected to increase steeply in the upcoming decades. Unfortunately, there is currently no cure for PD, with available medications only providing symptomatic relief.^3^

PD is a movement disorder clinically characterized by tremor, bradykinesia, rigidity, and postural instability.^4^ The motor dysfunctions are a direct consequence of the death of dopamine-producing neurons in the substantia nigra pars compacta region of the midbrain. Histologically, the most noticeable feature of PD are abnormal aggregates of proteins, called Lewy bodies and Lewy neurites, that appear in the cell body and neurites of PD patient neurons. Their major constituent is the protein alpha-synuclein.

Familial genetic linkage studies have unequivocally associated six genes with Mendelian inheritable forms of PD.^4^ Still, these individual gene mutations account for fewer than 10% of PD cases. They generally lead to juvenile or early onset PD (before 50 years of age). Naturally, genetics still impacts the risk of an individual acquiring non-monogenic sporadic PD later in life. Over a dozen single nucleotide polymorphisms (SNPs) have been statistically linked with sporadic PD through genome-wide association studies (GWASs).^5^,^6^ However, the differential risks associated with carrying these SNPs, although statistically significant, are mostly very small in absolute terms. Similarly, although some environmental factors, such as exposure to metals or pesticides, have been statistically linked with PD, the associations do not appear to be sufficiently widespread to explain beyond a minority of PD cases.^7^ Thus, the etiology of the over 90% of cases classified as sporadic PD remains undetermined.

We briefly highlight some of the major theories being pursued regarding the nature of sporadic PD. Different aspects of these hypotheses will be presented in more detail as relevant, throughout the article. An overarching hypothesis on the etiology of sporadic PD is that it is triggered by an external agent. Pesticides and metals would be two candidate environmental agents, given their statistical association with PD.^7^ Another proposed factor suggested in the literature is a neurothropic pathogen, such as a virus^8^ or a prion-like protein.^9^ Entry into the organism could be via the peripheral olfactory system^10^ or via the gastrointestinal tract,^11^ two sites associated with early prodromal clinical symptoms of PD. These two sites have also been combined into a dual-hit hypothesis, centered on an external agent simultaneously entering the organism via the two routes.^12^ Whether with an initially external origin or endogenously generated, the theory that a misfolded, prion-like self-propagating form of alpha-synuclein is responsible for the disease is another major hypothesis currently under investigation.^9^,^13^ More endogenous, aging-related perspectives of sporadic PD focus on the role of oxidative stress and mitochondrial damage, for which there is significant evidence in PD patients. 14,15 Finally, although the view of sporadic PD as an autoimmune disease is not typical, the aggravating contribution of the neuro-inflammatory response to the disease is commonly acknowledged.^16^,^17^

Starting from the analysis of sporadic PD blood gene-expression data, we have previously argued that sporadic PD could have a hematopoietic origin.^18^ In the present article, we review and expand on this original hypothesis, placing it in the context of recent developments in both PD and the broader biological research. The article first addresses why sporadic PD may be a systemic, rather than solely neuronal, condition. Then, it discusses why inter-cellular propagation of the pathology may be involved, rather than the pathology being purely associated with cell ageing phenomena. In particular, we present the possibility that the disease transmission may be enabled by the inter-cellular exchange of the mitochondrial DNA. Finally, the article revisits our previously published hypothesis that the pathology may initially begin at hematopoiesis.

## The universal nature of the PD-state

In this section, we argue for the systemic nature of sporadic PD. Our case is centered on the interpretation of recently published evidence obtained by applying induced pluripotent stem cell (iPSC) technology to PD research.^19^,^20^ Using iPSC techniques, conveniently collected cells from PD patients, such as skin fibroblasts, can be reverted to a pluripotent state and subsequently differentiated into d opaminergic neurons. We believe that early PD iPSC experimental results support a fundamental re-evaluation of the nature of the sporadic non-monogenic form of the disease.

We summarize the main observations from two of the published PD iPSC studies.^19^,^20^ In both studies, skin fibroblasts from sporadic PD patients (presuppose sporadic henceforth) were reprogrammed back into pluripotent stem cells, which were then differentiated into dopaminergic neurons. Sánchez-Danés et al.^19^ reported that, in comparison with neurons derived from fibroblasts from disease-free controls, neurons originating in fibroblasts from PD patients consistently showed PD phenotype associated alterations. These included reduced numbers of neurites, more limited neurite arborizations, and increases in caspase-3 activity, a marker for cell apoptosis. Woodard et al.^20^ utilized neurons derived from fibroblasts of two monozygotic twins discordant for PD. Multiple alterations that can be linked with a PD phenotype were present exclusively in the fibroblasts derived from the PD twin. These included lower dopamine levels, an elevation of alpha-synuclein in neurites, a delay in the emergence of spontaneous action potentials, and an absence of synchronous neuronal activity.

An epidemiological study by Tanner et al.^21^ reported a mere 15.5% concordance of monozygotic twins in developing PD. An analogous study in Sweden published by Wirdefeldt et al.^22^ corroborated this result, placing the concordance rate at 11%. Thus, excluding the monogenic cases, heredity does not ensure the emergence of PD. Therefore, the consistent, regular emergence of a PD phenotype in neurons derived from fibroblasts from PD patients cannot be attributed to a PD favorable germline genetic background in the patients. The conclusion is that the disease must have been present in the skin fibroblasts from the patients. PD is thus a systemic condition, not confined to neuronal cells.

We propose to characterize PD as a pathological deviation in the expression program of a cell: the PD expression-state, or PD-state for short.^18^,^23^ Reports of a characteristic PD gene-expression signature across multiple tissues support this view.^18^,^24^–^26^ The recent observation of a unique, concordant pattern of methylation in post-mortem frontal cortex samples and peripheral blood leukocytes from PD patients^27^ reinforces this standpoint, further suggesting that the PD-state may be stabilized by DNA epigenetic modifications.

## The role of ageing in PD

In spite of possessing a multitude of self-repair mechanisms, all cells undergo the ageing process.^28^ They gradually accumulate dysfunctional molecules, as well as random mutations and other assorted alterations in their genetic code, ultimately resulting in the ageing phenotype. As a source of free radicals, mitochondria and the mitochondrial DNA are particularly vulnerable to oxidative stress damage.^29^ This observation has led to the theory that the mitochondrial dysfunction caused by oxidative stress plays a central role in ageing.^30^,^31^ With PD arising at old age and with mitochondrial function specifically known to be compromised in a variety of cell types in PD patients,^32^–^35^ the mitochondrial theory of aging broadly views PD as yet another manifestation of this phenomenon.^14^

It is conceivable that accumulated random damage, possibly in mitochondrial DNA and due to oxidative stress, eventually triggers the gene expression program of a cell to shift to the PD-state. This shift to the PD-state in a cell could thus be viewed as a probabilistic event, its likelihood being a (nonlinearly) increasing function of the accumulated damage. However, we argue that it is impossible for all the PD-state cells in an affected individual to have acquired the PD-state in this fashion.

The following valid scenario may be considered. Imagine two monozygotic twins, one diagnosed with PD in the past, with the other being disease-free at the present time. Let the unaffected twin presently exhibit a greater amount of age accumulated damage than the affected twin did at the time of his PD diagnosis – this would be the case given enough years had elapsed since the original diagnosis. Now, assume that accumulated random damage is the only possible trigger of the PD-state in a cell. Then, the observed greater age accumulated damage in the undiagnosed twin at present would guarantee a currently greater probability for his or her individual cells to acquire the PD-state than that probability was for the cells of the affected twin at the time of his PD-diagnosis. Yet, the unaffected twin continues to show no clinical signs of PD at present, in contrast with the affected twin at the time of his PD diagnosis. Thus, assuming that accumulated random damage is the only possible trigger of the PD-state in a cell leads to a probabilistic paradox.

We make two remarks on the argument presented in the previous paragraph. Firstly, accumulated random damage causing a cell to shift to the PD-state does not pose a paradox. Only the presence of most of the PD-state cells in the organism cannot be explained by such a process. Therefore, ageing-associated damage, caused by oxidative stress in mitochondria, could still be the trigger for a primordial cell in the organism to shift to the PD-state. Secondly, ageing-associated cellular damage may not be required for the subsequent dissemination of the PD-state across the organism since, as argued, this dissemination would occur via a distinct mechanism.

There are two direct pieces of evidence supporting a propagation dynamic in PD. First, analyses of post-mortem neuronal tissue from patients who died at different stages of the disease appear to support a chronological, physical spread of Lewy-bodies across the nervous system.^36^ Second, there is published evidence on the surgical transplantations of fetal ventral mesencephalic dopaminergic neurons as a treatment of PD. A number of post-mortem analyses, performed over 10 years after the transplant, detected Lewy-bodies and Lewy neurites in grafted neurons, in spite of the still young age of the transplanted tissue.^37^,^38^ This latter fact is in addition evidence of the non-essentiality of cellular ageing to the acquisition of the PD-state.

In this section, we argued that ageing-associated damage caused by oxidative stress in mitochondria could be the trigger for a primordial individual cell to shift its gene expression program to the PD-state. The shift to the PD-state would be a probabilistic event, its likelihood increasing nonlinearly with that accumulated damage in the cell. On the other hand, the subsequent appearance of the PD-state in numerous other cells could no longer be explained by cell ageing. Rather, it likely involves a separate mechanism of propagation of the PD-state.

## The propagation of the PD-state

The spread of the PD-state is a slow process, as evidenced by the approximate decade that it takes an implanted fetal neuron in the brain of a PD patient to develop Lewy bodies.^37^,^38^ The mechanism of propagation of the PD-state remains undetermined. However, new modes of inter-cellular communication continue to be discovered.^39^ In this section, we discuss how propagation of the PD-state may occur.

One hypothesis posits that alpha-synuclein can behave as a prion and that PD is a prion disorder.^9^,^13^ Under this theory, there exists a misfolded form of alpha-synuclein that is self-propagating, having the ability to induce similar misfolding in well-conformed alpha-synuclein. The transfer of the misfolded form of alpha-synuclein from cell to cell would thus result in the inter-cellular spread of PD. However, a western blot analysis did not detect any alpha-synuclein in the fibroblasts utilized in the PD iPSC experiments discussed earlier.^20^ Therefore, a PD phenotype in fibroblast-derived neurons cannot be explained by the lingering presence of a hypothetical infectious form of alpha-synuclein.

Nevertheless, there is the possibility that a different biological entity is responsible for propagating the PD-state. Recently, the inter-cellular exchange of mitochondrial DNA (mtDNA) has been demonstrated.^40^,^41^ We propose that mtDNA could be the vehicle for the inter-cellular transmission of the PD-state.

Comprehensive research with neuron-platelet cytoplasmic hybrids supports that anomalous mtDNA may suffice to set off the PD-state in a cell. A PD cybrid cell is created in vitro by the fusion of a neuronal cell depleted of endogenous mtDNA with an enucleated platelet from a PD donor. Thus, the mtDNA of the cybrid cell is that of the platelet from the PD patient, while its nuclear DNA is that of the disease-free neuronal cell. Various PD characteristic alterations have been observed in PD cybrids, most prominently, inclusions that replicate the essential biochemical and structural features found in Lewy-bodies in the brain of PD patients.^15^,^42^,^43^

The sufficiency of mtDNA to trigger the PD-state in a cell is supported by its ability to induce epigenetic modifications and to modulate gene-expression in nuclear DNA. In the context of tumorigenesis, work by Smiraglia et al.^44^ and by Xie et al.^45^ shows that alterations to mtDNA affect the methylation pattern of various nuclear genes. Bellizzi et al.^46^ report that methylation and gene expression patterns of nuclear genes in cybrids depend on the mtDNA donor haplogroup. Kelly et al.^47^ proposed that mtDNA haplotypes play a pivotal role in the process of differentiation and mediate the fate of the cell. In mouse undifferentiated and differentiating embryonic stem cells, with the same nuclear DNA haplotype but distinct mtDNA haplotypes, they observed mtDNA haplotype-specific expression of genes involved in pluripotency, differentiation, mitochondrial energy metabolism, and DNA methylation.

No specific mutations in mtDNA have been consistently associated with PD.^48^ However, heteroplasmy of mtDNA (i.e., the presence of multiple mtDNA variants within a cell) is now widely appreciated.^41^ Thus, undetected lower frequency mtDNA variants could potentially be involved in the transmission of the PD-state. Additionally, the number of PD-state triggering variants could be too large for effective statistical detection. Alternatively, the PD-state could be a result of mtDNA epigenetic modifications. Whether mtDNA can be methylated is currently a matter of active debate.^49^,^50^ Hong et al.^51^ make a strong case for the absence of mtDNA methylation, at least under most biological conditions. On the other hand, a recent publication by Bacarelli et al.^52^ reports the presence of significant mtDNA methylation in platelets of cardiovascular disease patients. For instance, in sequenced sites in the MT-CO1 gene region, Bacarelli et al. report average percentage site methylation to be on the order of 25%.^52^

## A site of origin for the PD-state

We have mentioned that ageing-associated damage caused by oxidative stress in mitochondria could be the trigger for a primordial cell to shift its gene expression program to the PD-state. The next relevant question is: Where would an initial PD-state cell most commonly arise? In this section, we suggest the hematopoietic stem cell niche as a site to consider.

Research based on gene expression,^18^,^25^ DNA methylation,^27^ neuron-platelet cybrid,^15^,^42^,^43^ and bioenergetic^33^ analyses supports the presence of the PD-state in circulating hematopoietic cells. Given the short lifespan of blood cells (days for platelets^53^ and granulocytes^54^ and weeks for lymphocytes, with the exception of memory cells^55^) by comparison with the decade long timescale for the transmission of PD across the neuronal system,^36^–^38^ the above signs of PD in blood point to circulating hematopoietic cells acquiring the PD-state at hematopoiesis, rather than after maturation.

Hematopoiesis is altered with ageing. In terms of global gene expression in hematopoietic stem cells (HSCs), nitric oxide mediated signal transduction, the NF-kB cascade and the pro-inflammatory response are the most age up-regulated processes, while chromatin silencing, single-strand break repair, SMAD protein nuclear translocation and chromatin remodeling are the most down-regulated ones.^56^ Alterations at the HSC epigenetic level are supported by many chromosomal regions showing a coordinated change in transcriptional activity.^56^ Fate-wise, a skewing to the myeloid line and a diminished lymphoid potential are observed with ageing.^57^

It has been known for a long time that reactive oxygen species (ROS), if not properly checked, have the potential to cause indiscriminate cellular damage.^30^ Today, it is recognized that ROS may also play a functional signaling role in activating processes such as the inflammatory and stress responses.^28^,^58^ Additionally, there is indication that ROS play a role in regulating hematopoiesis.^59^ In particular, evidence associates abnormal ROS levels at old age with a dysfunction in both the proliferation and the differentiation dynamics of HSCs. In vitro research has shown that exposure to H_2_O_2_ can lead to chromosomal translocations in HSCs.^60^ Ionizing radiation is also known to affect HSCs, as it has been found to promote differentiation, short-term apoptosis and long-term senescence of HSCs.^61^ Work with Drosophila supports the role of ROS in the regulation of hematopoietic cell fate. Increasing ROS beyond its basal level in Drosophila multipotent hematopoietic progenitor cells triggers their precocious differentiation.^62^ Conversely, scavenging ROS from these hematopoietic progenitors retards their differentiation into mature blood cells.^62^ It is well-established that serial transplantation of human HSCs into immunodeficient mice leads to both elevated intracellular ROS levels and to premature HSC senescence.^63^–^65^ Yahata et al.^63^ and Ito et al.^65^ independently reported that antioxidant pharmacological inhibition of ROS can mitigate this deteriorating HSC phenotype. Caloric restriction in BalbC mice was similarly shown to postpone HSC senescence.^64^ Finally, the same protective effect was achieved by SIRT3 up-regulation in HSCs.^66^ HSCs are highly-enriched in this mammalian sirtuin, except for its suppression at old age.^66^ SIRT3 regulates the global acetylation landscape of mitochondrial proteins and reduces oxidative stress.^66^ Mechanistically, the FoxO transcription factors^67^ and the p53,^68^ Akt,^69^ MAPK,^65^ and ATM^70^ pathways have all been implicated in the ROS modulation of hematopoiesis.

At the genetic level, emerging evidence may also connect PD and the hematopoietic system, although its interpretation is not yet completely clear. A new study by Xiao et al.^71^ found hematologic abnormalities in alpha-synuclein knock-out mice indicative of a role of alpha-synuclein in late-stage hematopoiesis. A genome-wide association study found that a rare non-synonymous mutation in DZIP1 is a risk factor for PD.^72^ DZIP1 is a component of the Hedgehog signaling pathway.^73^ Besides its role in directing embryonic pattern formation, the hedgehog pathway has been implicated in the maintenance of adult stem cell niches, including both neuronal^74^ and hematopoietic stem-cells.^75^ Finally, PD patients are over five times more likely to be carriers of the mutated form of GBA responsible for the Gaucher’s autosomal recessive disease.^76^ Gaucher’s disease is characterized by low blood platelet levels, anemia, and the accumulation of the glycolipid glucocerebroside in the mononuclear phagocyte system.^77^

In summary, we argued for the hematopoietic stem cell niche as a possible site for the appearance of a primordial PD-state cell based on evidence that supports: i) PD patients consistently having circulating hematopoietic cells in the PD-state; ii) Those hematopoietic cells more plausibly having acquired the PD-state at hematopoiesis, rather than after maturation; and iii) The critical role of ROS in regulating the hematopoietic niche and the disruption of this homeostasis with ageing.

## Conclusion

Based on recently published findings that we considered relevant to the PD field, as well as on various established lines of PD research, we presented a comprehensive theory on the nature, origin, and inter-cellular mode of propagation of sporadic Parkinson’s disease. We propose to define PD as a characteristic pathological deviation in the global gene expression program of a cell: the PD expression-state, or PD-state for short. Most significantly, we argue that any cell could be in the PD-state. However, due to the cell processes it affects the most, the PD-state deviation would be particularly damaging to neurons, ultimately leading to neuron death and the clinical manifestations of PD.

Ageing-associated accumulated damage caused by oxidative stress in mitochondria could be the trigger for a primordial cell to shift to the PD-state. In particular, hematopoietic cells could be the first to acquire the PD-state, at hematopoiesis, as a result of the disruption in ROS homeostasis that arises with age in the hematopoietic stem-cell niche. The small correlation of PD incidence with genetic and environmental factors would mostly follow from this PD initiation dynamics.

Propagation of the PD-state across the organism would occur in a second phase and via a distinct mechanism. We proposed that the ability of mtDNA to move across cells and to modulate the cellular global gene expression state could form the basis for this inter-cellular propagation of the PD-state. The mtDNA-based PD propagation dynamics would occur on a time-scale of years, as observed in patients, and not be ageing-dependent, in contrast with the PD initiation dynamics.

Under physiological conditions, mice are not susceptible to PD late in life, in spite of clearly showing an ageing phenotype just as humans do.^78^ This absence of a PD phenotype in mice could thus more likely follow from the ageing-independent PD spread dynamics, than from the ageing-associated PD initiation dynamics. Namely, going from human to mouse, the mtDNA propagation dynamics would not scale appropriately time-wise to permit the condition to reach the neuronal system in the lifetime of a mouse.

Early symptoms of PD in humans include impaired sense of smell^10^ and gastrointestinal dysfunction.^79^ Both have been reported as much as a decade before the appearance of symptoms at the motor level. They are typically interpreted as supporting the role of an external agent - entering via the olfactory or gastro-intestinal entry points - in inducting PD.^12^ However, another characteristic shared by the olfactory bulb and the gastro-intestinal tract is that they are both sites of very active stem-cell based tissue regeneration.^80^,^81^ The rapid cell renewal and the plasticity of immature cells could facilitate both the cellular uptake of carriers of external mtDNA and the global cellular reprogramming to the PD-state, explaining the olfactory bulb and the gastro-intestinal tract being some of the earlier sites to which the PD-state would spread.

Finally, although our theory was presented in the context of PD, we note its general potential relevance to other pathologies where systemic bioenergetic cellular deficiency is a prominent feature.

## References

[1] Statistics on Parkinson’s.

[2] Shrestha LB (2000). Population aging in developing countries.. Health Aff.

[3] Davie CA (2008). A review of Parkinson’s disease..

[4] Beitz JM (2014). Parkinson’s disease: A review.. Front Biosci.

[5] (2011). Imputation of sequence variants for identification of genetic risks for Parkinson’s disease: a meta-analysis of genome-wide association studies.. Lancet.

[6] Nalls MA, Pankratz N, Lill CM (2014). Large-scale meta-analysis of genome-wide association data identifies six new risk loci for Parkinson’s disease.. Nat Genet.

[7] Monte DAD, Lavasani M, Manning-Bog AB (2002). Environmental factors in Parkinson’s disease.. Neurotoxicology.

[8] Takahashi M, Yamada T (1999). Viral etiology for Parkinson’s disease - a possible role of influenza A virus infection.. Jpn J Infect Dis.

[9] Olanow CW, Prusiner SB (2009). Is Parkinson’s disease a prion disorder?. Proc Natl Acad Sci US A.

[10] Lerner A, Bagic A (2008). Olfactory pathogenesis of idiopathic Parkinson disease revisited.. Mov Disord.

[11] Phillips RJ, Walter GC, Wilder SL, Baronowsky EA, Powley TL (2008). Alpha-synucleinimmunopositive myenteric neurons and vagal preganglionic terminals: autonomic pathway implicated in Parkinson’s disease?. Neuroscience.

[12] Hawkes CH, Del Tredici K, Braak H (2007). Parkinson’s disease: a dual-hit hypothesis.. Neuropath Appl Neuro.

[13] Desplats P, Lee H-J, Bae E-J, Patrick C, Rockenstein E, Crews L (2009). Inclusion formation and neuronal cell death through neuron-to-neuron transmission of α-synuclein..

[14] Henchcliffe C, Beal MF (2008). Mitochondrial biology and oxidative stress in Parkinson disease pathogenesis.. Nat Clin Pract Neuro.

[15] Swerdlow RH (2012). Does Mitochondrial DNA Play a Role in Parkinson’s Disease? A Review of Cybrid and Other Supportive Evidence.. Antioxid Redox Signal.

[16] Monahan AJ, Warren M, Carvey PM (2008). Neuroinflammation and Peripheral Immune Infiltration in Parkinson’s Disease: An Autoimmune Hypothesis.. Cell Transplant.

[17] Whitton P (2007). Inflammation as a causative factor in the aetiology of Parkinson’s disease.. Br J Pharmacol.

[18] Valente AXCN, Sousa JAB, Outeiro TF, Ferreira L, Valente AXCN (2011). A stem-cell ageing hypothesis on the origin of Parkinson’s disease.. Science and engineering in high-throughput biology including a theory on Parkinson’s disease.

[19] Sánchez-Danés A, Richaud-Patin Y, Carballo-Carbajal I (2012). Disease-specific phenotypes in dopamine neurons from human iPS-based models of genetic and sporadic Parkinson’s Disease.. EMBO Mol Med.

[20] Woodard CM, Campos BA, Kuo S-H (2014). iPSC-Derived dopamine neurons reveal differences between monozygotic twins discordant for Parkinson’s disease.. Cell Reports.

[21] Tanner CM, Ottman R, Goldman SM (1999). Parkinson disease in twins: an etiologic study.. JAMA - J Am Med Assoc.

[22] Wiredefeldt K, Gatz M, Reynolds CA, Prescott CA, Pedersen L (2011). Heritability of Parkinson disease in Swedish twins: a longitudinal study.. Neurobiol Aging.

[23] Valente AXCN, Oliveira PJ, Khaiboullina SF, Palotás A, Rizvanov AA (2013). Biological Insight, High-Throughput Datasets and the Nature of Neuro-Degenerative Diseases.. Curr Drug Metab.

[24] Simunovic F, Yi M, Wang Y (2009). Gene expression profiling of substantia nigra dopamine neurons: further insights into Parkinson’s disease pathology.. Brain.

[25] Scherzer CR, Eklund AC, Morse LJ (2007). Molecular markers of early Parkinson’s disease based on gene expression in blood.. Proc Natl Acad Sci U S A.

[26] Mandel S, Grunblatt E, Riederer P (2008). Gene Expression Profiling of Sporadic Parkinson’s Disease Substantia Nigra Pars Compacta Reveals Impairment of Ubiquitin-Proteasome Subunits, SKP1A, Aldehyde Dehydrogenase, and Chaperone HSC-70.. Ann N Y Acad Sci.

[27] Masliah E, Dumaop W, G D, Desplats P (2013). Distinctive patterns of DNA methylation associated with Parkinson disease.. Epigenetics.

[28] Lane N (2003). A unifying view of ageing and disease: the double-agent theory.. J Theor Biol.

[29] Murphy MP (2009). How mitochondria produce reactive oxygen species.. Biochem J.

[30] Harmann D (1956). Aging - a theory based on free-radical and radiation chemistry.. J Gerontol.

[31] Barja G (2013). Updating the mitochondrial free-radical theory of aging: an integrated view, key aspects, and confounding concepts.. Antioxid Redox Signal.

[32] Wiedemann FR, Winkler K, Lins H, Wallesch C-W, Kunz WS (2006). Detection of respiratory chain defects in cultivated skin fibroblasts and skeletal muscle of patients with Parkinson’s disease.. Ann N Y Acad Sci.

[33] Barroso N, Campos Y, Huertas R, Esteban J, Molina JA (1993). Respiratory chain enzyme activities in lymphocytes from untreated patients with Parkinson disease.. Clin Chem.

[34] Yoshino H, Nakagawa-Hattori Y, Kondo T, Mizuno Y (1992). Mitochondrial complex I and II activities of lymphocytes and platelets in Parkinson’s disease.. J Neural Transm.

[35] Mytilineou C, Werner P, Molinari S, Rocco AD, Cohen G, Yahr MD (1994). Impaired oxidative decarboxylation of pyruvate in fibroblasts from patients with Parkinson’s disease.. J Neural Transm.

[36] Braak H, Tredici KD, Rüb U, Vos RAId, Steur ENHJ, Braak E (2003). Staging of brain pathology related to sporadic Parkinson’s disease.. Neurobiology Aging.

[37] Li JY, Englund E, Holton JL (2008). Lewy bodies in grafted neurons in subjects with Parkinson’s disease suggest host-to-graft disease propagation.. Nat Med.

[38] Kordower JH, Chun Y, Hauser RA, Freeman TB, Olanow CW (2008). Lewy body-like pathology in long-term embryonic nigral transplants in Parkinson’s disease.. Nat Med.

[39] Valadi H, Ekström K, Bossios A, Sjöstrand M, Lee JJ (2007). Exosome-mediated transfer of mRNAs and microRNAs is a novel mechanism of genetic exchange between cells.. Nat Cell Biol.

[40] Tan AS, Baty JW, Dong L-F (2015). Mitochondrial genome acquisition restores respiratory function and tumorigenic potential of cancer cells without mitochondrial DNA.. Cell Metab.

[41] Jayaprakash AD, Benson EK, Gone S (2015). Stable heteroplasmy at the single-cell level is facilitated by intercellular exchange of mtDNA.. Nucleic Acids Res.

[42] Trimmer PA, Borland MK, Keeney PM, B JP, P WD (2004). Parkinson’s disease transgenic mitochondrial cybrids generate Lewy inclusion bodies..

[43] Esteves AR, Domingues AF, Ferreira IL (2008). Mitochondrial function in Parkinson’s disease cybrids containing an nt2 neuron-like nuclear background.. Mitochondrion.

[44] Smiraglia DJ, Kulawiec M, Bistulfi GL, Gupta SG, Singh KK (2008). A novel role for mitochondria in regulating epigenetic modification in the nucleus.. Cener Biol Ther.

[45] Xie CH, Naito A, Mizumachi T, Evans TT, Douglas MG, Cooney CA (2007). Mitochondrial regulation of cancer associated nuclear DNA methylation.. Biochem Biophys Res Commun..

[46] Bellizzi D, D’Aquila P, Giordano M, Montesanto A, Passarino G (2012). Global DNA methylation levels are modulated by mitochondrial DNA variants.. Epigenomics.

[47] Kelly RD, Rodda AE, Dickinson A (2013). Mitochondrial DNA haplotypes define gene expression patterns in pluripotent and differentiating emrbyonic stem cells.. Stem Cells.

[48] Schapira AHV (2008). Mitochondria in the aetiology and pathogenesis of Parkinson’s disease.. Lancet Neurol.

[49] Maresca A, Zaffagnini M, Caporali L, Carelli V, Zanna C DNA methyltransferase 1 mutations and mitochondrial pathology: is mtDNA methylated?. Front Genet.

[50] Ghosh S, Singh KK, Sengupta S, Scaria V (2015). Mitoepigenetics: the different shades of grey.. Mitochondrion.

[51] Hong EE, Okitsu CY, Smith AD, Hsieh CL (2013). Regionally specific and genome-wide analyses conclusively demonstrate the absence of CpG methylation in humann mitochondrial DNA.. Mol Cell Biol.

[52] Bacarelli AA, Byun HM (2015). Platelet mitochondrial DNA methylation: a potential new marker of cardiovascular disease.. Clinical Epigenetics.

[53] Stuart MJ, Murphy S, Oski FA (1975). A simple nonradioisotope technic for the determination of platelet life-span.. N Engl J Med.

[54] Simon SI, Kim MH (2010). A day (or 5) in a neutrophil’s life.. Blood.

[55] Tough DF, Sprent J (1995). Lifespan of lymphocytes.. Immunol Res.

[56] Chambers SM, Shaw CA, Gatza C, Fisk CJ, Donehower LA, Goodell MA (2007). Aging hematopoietic stem cells decline in function and exhibit epigenetic dysregulation.. PLoS Biol.

[57] Rossi DJ, Bryder D, Zahn JM (2005). Cell intrinsic alterations underlie hematopoietic stem cell aging.. Proc Natl Acad Sci USA.

[58] Hamanaka RB, Chandel NS (2010). Mitochondrial reactive oxygen species regulate cellular signaling and dictate biological outcomes.. Trends Biochem Sci.

[59] Pervaiz S, Taneja R, Ghaffari S (2009). Oxidative stress regulation of stem and progenitor cells.. Antioxid Redox Signal.

[60] Francis R, Richardson C (2007). Multipotent hematopoietic cells susceptible to alternative double-strand break repair pathways that promote genome rearrangements.. Genes Dev.

[61] Shao L, Luo Y, Zhou D (2014). Hematopoietic stem-cell injury induced by ionizing radiation.. Antioxid Redox Signal.

[62] Owusu-Ansah E, Banerjee U (2009). Reactive oxygen species prime drosophila hematopoietic progenitors for differentiation.. Nature.

[63] Yahata T, Takanashi T, Muguruma Y (2011). Accumulation of oxidative DNA damage restricts the self-renewal capacity of human hematopoietic stem cells.. Blood.

[64] Chen J, Astle CM, Harrison DE (2003). Hematopoietic senescence is postponed and hematopoietic stem cell function is enhanced by dietary restriction.. Exp Hematol.

[65] Ito K, Hirao A, Arai F (2006). Reactive oxygen species act through p38 MAPK to limit the lifespan of hematopoietic stem cells.. Nat Med.

[66] Brown K, Xie S, Qiu X (2013). SIRT3 reverses aging-associated degeneration.. Cell Rep.

[67] Tothova Z, Kollipara R, Huntly BJ (2007). FoxOs are critical mediators of hematopoietic stem cell resistance to physiologic oxidative stress.. Cell.

[68] Abbas HA, Maccio DR, Coskun S, Jackson JG, Hazen AL, Sills TM (2010). Mdm2 is required for survival of hematopoietic stem cells/progenitors via dampening of ROS-induced p53 activity.. Cell Stem Cell.

[69] Yalcin S, Marinkovic D, Mungamuri SK (2010). ROS-mediated amplification of AKT/mTOR signalling pathway leads to myeloproliferative syndrome in Foxo3−/− mice.. EMBO J.

[70] Ito K, Hirao A, Arai F (2004). Regulation of oxidative stress by ATM is required for self-renewal of haematopoietic stem-cells.. Nature.

[71] Xiao W, Shameli A, Harding CV, Meyerson HJ, Maitta RW (2014). Late stages of hematopoiesis and B cell lymphopoiesis are regulated by α-synuclein, a key player in Parkinson’s Disease.. Immunobiology.

[72] Valente AXCN, Shin JH, Sarkar A, Gao Y Rare coding SNP in DZIP1 gene associated with late-onset sporadic Parkinson’s disease.. Sci Rep.

[73] Sekimizu K, Nishioka N, Sasaki H, Takeda H, Karlstrom RO, Kawakami A (2004). The zebrafish iguana locus encodes Dzip1, a novel zinc-finger protein required for proper regulation of Hedgehog signaling..

[74] Palma V, Lim DA, Dahmane N, Sánchez P, Brionne TC (2004). Sonic hedgehog controls stem cell behavior in the postnatal and adult brain.. Development.

[75] Bhardwaj G, Murdoch B, Wu D, Baker DP, Williams KP (2001). Sonic hedgehog induces the proliferation of primitive human hematopoietic cells via BMP regulation.. Nature Immunol.

[76] Sidransky E, Nalls MA, Aasly JO (2009). Multicenter Analysis of Glucocerebrosidase Mutations in Parkinson’s Disease.. N Engl J Med.

[77] Mingyi C, Jun W (2008). Gaucher Disease: Review of the Literature.. Arch Pathol Lab Med.

[78] Dawson TM (2000). New Animal Models for Parkinson’s Disease.. Cell.

[79] Natale G, Pasquali L, Ruggieri SAP, Fornai F (2008). Parkinson’s disease and the gut: a well known clinical association in need of an effective cure and explanation.. Neurogastroenterol Motil.

[80] Creamer B, Shorter RG, Bamforth J (1961). The turnover and shedding of epithelial cells. I. The turnover in the gastro-intestinal tract.. Gut.

[81] Mouret A, Lepousez G, Gras J, Gabellec MM, Lledo PM (2009). Turnover of newborn olfactory bulb neurons optimizes olfaction.. J Neurosci.

